# Sleep, time, and space—fatigue and performance deficits in pilots, commercial truck drivers, and astronauts

**DOI:** 10.1093/sleepadvances/zpac033

**Published:** 2022-09-15

**Authors:** Katherine A Maki, Anne M Fink, Terri E Weaver

**Affiliations:** Translational Biobehavioral and Health Disparities Branch, Clinical Center, National Institutes of Health, Bethesda, MD, 20814, USA; Department of Biobehavioral Nursing Science, College of Nursing, University of Illinois Chicago, Chicago, IL, 60612, USA; Department of Biobehavioral Nursing Science, College of Nursing, University of Illinois Chicago, Chicago, IL, 60612, USA; Department of Biobehavioral Health Sciences, School of Nursing, University of Pennsylvania, Philadelphia, PA, 19104, USA

**Keywords:** accidents, aviation, driving, psychomotor vigilance tests, occupational health, safety, shift-work

## Abstract

Sleep is essential for preventing fatigue in occupations that require sustained vigilance. We conducted a scoping review to synthesize knowledge about sleep, fatigue, and performance in pilots, commercial truck drivers, and astronauts. We found 28 studies where researchers objectively or subjectively measured sleep, fatigue, and performance. The research included laboratory-based (simulator) and field-based studies (i.e. real-world missions and a variety of shift-work schedules). Most researchers used actigraphy to measure sleep, and they found that ~6 hrs of sleep was common. The research also demonstrated how sleep duration and quality were negatively affected by schedule irregularity, early-morning start times, and high-risk missions (e.g. extravehicular activities in space). Collectively, the data demonstrated how shorter sleep durations, short off-duty time, and early-morning start times were associated with slower reaction times, more lapses in attention, and premature responses on psychomotor vigilance tests. Considering that few studies included polysomnography and circadian rhythm biomarkers, there remains limited knowledge about the effects of sleep microstructure and circadian rhythm alterations on performance abilities in these occupations. Future neurobiological and mechanistic discoveries will be important for enhancing vigilance, health, and safety for people working in the skies, on the roads, and in space.

This paper is part of the David F. Dinges Festschrift Collection. This collection is sponsored by Pulsar Informatics and the Department of Psychiatry in the Perelman School of Medicine at the University of Pennsylvania.

**Statement of Significance**

Fatigue can have devastating consequences for people in sleepiness-sensitive occupations, such as pilots, truck drivers, and astronauts. Our scoping review highlights important findings about the variables that affect sleep, fatigue, and vigilance in these occupations in addition to illustrating the importance of developing interventions that could mitigate fatigue. Future research will be important for determining how alterations in circadian rhythms and sleep architecture affect job performance.

## Introduction

Sleep is critical for maintaining physical and psychological health. Adults require ~7 hrs of sleep each day, according to the American Academy of Sleep Medicine and the Sleep Research Society [1]. From an occupational health perspective, fatigue (a biological demand for recuperative rest) threatens safety and job performance [2]. Many highly publicized accidents have been attributed to fatigue, which underscores the importance of understanding how to promote healthy sleep and workplace/public safety [2]. Occupational responsibilities may restrict the times people have available to sleep. In the transportation industries, for example, people often work across multiple times zones, which can cause their circadian rhythms to become misaligned with their schedules [3]. In occupations that require nighttime work, people rely on their abilities to obtain enough daytime sleep, although this is opposed by humans’ natural sleep–wake cycle patterns that favor wakefulness during the daytime and sleep during the night [4–7]. Work-related stress and environmental conditions (e.g. uncomfortable sleeping locations, sustained light exposure, and noise) also disrupt sleep and increase the risk for poor performance during work due to fatigue [2, 8, 9].

Airline pilots, commercial truck drivers, and astronauts are examples of occupations that require high-quality sleep for sustained vigilance during work. To perform their jobs effectively, they must endure stressful conditions and combine astute cognitive abilities with technical knowledge to make decisions safely under rapidly changing conditions [10–19]. Research has demonstrated how insufficient sleep impairs work-time functioning [12, 20, 21], which ultimately led to regulations that enforced duty restrictions and recommended fatigue-management strategies. Examples include Title 14 of the Code of Federal Regulations, which outlined duty limitations for safer aviation in 2014 [22]; the U.S. Federal Motor Carrier Safety Administration’s 2014 revisions to duty time for truck drivers [23]; and the International Civil Aviation Organization’s 2015 fatigue-management guidelines [24].

Safety-focused regulations were driven by the scientific discoveries of Dinges et al. [10, 12, 25, 26], which established a battery of neurobehavioral performance metrics. Dinges and colleagues developed, tested, and validated methods for measuring vigilance and alertness, such as the 10-min Psychomotor Vigilance Test (PVT) [27, 28] 3-min Brief PVT (PVT-B) [27, 29, 30], and Cognition Test Battery [31]. The PVT and PVT-B have been valuable for objectively quantifying responses to randomly timed visual stimuli to improve the rigor of occupational health research (compared with relying on methods that could be confounded by learning effects or subjectivity) [32, 33]. Research participants’ subjective descriptions of fatigue, for example, may not be consistently correlated with the objective measurements provided by the PVT.

In occupations where sleep times are restricted and there is a high-risk for catastrophic accidents, people are highly trained/experienced, but they are not impervious to the adverse effects of attention lapses caused by inadequate sleep. Therefore, it is important for researchers to determine how sleep-related variables affect people’s abilities for safe and effective job performance. Ultimately, the goal will be the development of optimal workplace practices that promote healthy sleep and mitigate the risks for errors and accidents. Previous reviews on this topic have focused on sleep deprivation rather than the occupationally relevant phenomena of short sleep and circadian misalignment [32, 34–36]. In addition, earlier reviews did not provide information about multiple occupations to allow for comparisons to understand the complex relationships among work schedules, sleep, fatigue, and performance in different settings. Therefore, the purpose of the present paper was to conduct a scoping review to synthesize research findings about objective and subjective measures of sleep, fatigue, and performance from three occupations—pilots, truck drivers, and astronauts.

## Methods

A *scoping review* is a knowledge-synthesizing literature review that is guided by an exploratory question to determine key concepts, types of evidence, and research gaps [37, 38]. We conducted our review according to Arksey and O’Malley’s scoping review methodology [38]. The following question guided the review: *What is known about sleep, fatigue, and performance in the safety-sensitive occupations involving aviation, truck driving, and space missions?* We chose to examine multiple occupations to determine similarities and differences; this aspect of the scoping review was important for understanding whether findings were occupation-specific or common across professions.

To identify relevant research studies, we searched MEDLINE/PubMed in August 2022 (excluding papers published before 2014 because several organizations implemented duty restrictions to increase sleep around this time) [22–24, 39]. The keywords for the search were “sleep, circadian, fatigue, and performance” in combination with “airline pilots” (retrieved 12 citations), “truck drivers” (retrieved 4 citations), or “astronauts” (retrieved 5 citations). We screened the abstracts to exclude review papers and focus only on original research reports; ultimately, we selected 11 data-based papers about airline pilots (excluded 1 review), 3 about truck drivers (excluded 1 review), and 5 about astronauts. Additional articles were found by handsearching and examining article reference lists (leading to the addition of 6 additional papers about airline pilots and 3 papers about astronauts). A total of 28 research papers were used for the scoping review (Table 1).

**Table 1.** Study characteristics, measures, and key findings

Study citations and aims for the 28 reviewed articles	Sample size (% women) Age (years, mean ± SD)	Occupation, location (field or simulator), and work schedule	Measures of sleep, fatigue, and/or performance (objective and subjective)	Circadian rhythm-related findings	Sleep, fatigue, and performance findings
1. Alzehairi et al. [40] **Aim**: To screen pilots for sleep disorders, fatigue, and depression and determine risk factors	344 (% women not reported) 40 ± 8	***Airline pilots*** (short-haul) **Field** (Saudi Arabia) Duty schedules were defined as day, night, or both 53% of the pilots were First Officers; 47% were Captains	**Sleep (subjective):** PSQI, Athens Insomnia Scale **Fatigue (subjective):** Epworth Sleepiness Scale, Fatigue Severity Scale, VAS Depressive symptoms were screened with the Patient Health Questionnaire. The Berlin Questionnaire was use for sleep apnea screening *No objective measures of sleep or performance were examined*	*Circadian rhythms* *were not examined*	*Sleep duration was not reported* Many pilots had poor-quality sleep (i.e. a third of the sample had PSQI global scores >5). Poor sleep quality, sleepiness, and insomnia were more common in pilots who flew during both days and nights; they had significantly higher PSQI global scores, higher Epworth Sleepiness Scale scores, and more positive answers on the Athens Insomnia Index Age and experience (years) were negatively associated with PSQI scores (*r* = −0.83, *p* < .001 and *r* = −0.61, *p* < .001, respectively) and with positive answers on the Athens Insomnia Severity Index Scale (*r* = −0.82, *p* < .001 and *r* = −0.63, *p* <.001, respectively)
2. Arsintescu et al. [41] **Aim**: To determine relationships among work load, performance, subjective fatigue, sleep duration, and flight duration	90 (8% women) 33 ± 8	***Airline pilots*** (short-haul) **Field** (United States) *Specific work schedules were not reported*	**Sleep (objective):** Actigraphy **Fatigue (subjective):** Samn-Perelli scale **Performance (objective):** PVT (at TOD) The NASA Task Load Index was used to measure workload (subjective)	*Circadian rhythms* *were not examined*	Sleep duration was 7.7 ± 1.7 hrs Sleep duration was positively associated with the NASA Task Load Index scores (*r* = 0.04, *p* < .05) and the mental (*r* = 0.04, *p* < .05) and frustration (*r* = 0.04, *p* < .05) sub-components. Samn-Perelli fatigue scores were positively associated with the effort and performance components of the NASA Task Load Index (*r* = 0.10, *p* < .01 and *r* = 0.10, *p* < .01, respectively) Mean raw NASA Task Load Index scores were associated with PVT lapses (*r* = 0.10, *p* < .01) and PVT response speed (*r* = −0.10, *p* < .01). Effort and performance (NASA Task Load Index) were positively associated with PVT lapses (*r* = 0.16, *p* < .01 and *r* =.06, *p* < .01) and negatively associated with PVT response speed (*r* = −0.20, *p* < .01; *r* = −0.06, *p* < .01)
3. Arsintescu et al. [42] **Aim**: To develop a protocol for 6-sulfatoxy-melatonin analysis (urine) and examine methods for measuring sleep, fatigue, and performance	44 (9% women) 31 ± 7	***Airline pilots*** (short-haul) **Field** (United States) Data were compared with a Baseline Block (5 days of mid- morning flights); the subsequent schedule involved four early flights (0500), four high-workload mid-day flights (1030), and four late flights (1600) that landed after midnight. Pilots were studied for 34 days	**Sleep (objective):** Actigraphy **Circadian (objective):** Urinary 6-sulfatoxymelatonin levels **Fatigue (subjective):** Samn-Perelli Scale; Epworth Sleepiness Scale **Performance (objective):** PVT	The circadian phase (determined by urinary 6-sulfatoxy-melatonin levels) was affected by pilots’ start times	Sleep duration decreased before early-morning take-offs compared with mid-day flights (7.4 ± 0.9 hrs versus 8.2 ± 0.9 hrs *p* < .01). The mean PSQI scores did not differ according to start times Samn-Perelli scale scores for early (4.0 ± 0.9, *p* < .001), mid-day (3.9 ± 0.9, *p* < .001) and late starts (3.9 ± 0.9, *p* < .001) revealed how each of these times were associated with more fatigue than mid-morning start times (3.5 ± 0.8) The PVT mean RT was significantly slower, and mean number of lapses was significantly higher for early, mid-day, and late start times (compared with the baseline period of mid-morning start times)
4. Barger et al. [43] **Aim**: To characterize sleep and hypnotic use during short and long missions and determine how chronic sleep-restriction affected performance	64 (16%women) 46 ± 4	***Astronauts*** **Laboratory** (Earth/United States within the 3 months before launch and for 1 week after the space mission) and **Field** (Space [for 1 to 30 weeks])	**Sleep (objective):** Actigraphy **Sleep and Fatigue (subjective):** Diaries, VAS for sleep quality and alertness Subjects also completed medication logs *No objective measures of performance were examined*	*Circadian rhythms were not examined*	Sleep duration 5.9 ± 0.6 hrs on the Space Transportation System. Astronauts assigned to the ISS demonstrated a reduction in sleep time 3 months and 11 days pre-flight; sleep was shortest while in space (5.9 ± 0.9 hrs, *p* < .0001compared with post-mission sleep) Sleep duration declined significantly after the first week of the mission. In space, 78% of crew members used hypnotics to sleep (versus 27% in the 3 months before launch, *p* < .0001) Astronauts slept <6 hrs on 51% of nights before EVAs. Average total sleep time was shortest before EVAs (5.9 ± 1.0 hrs) and sleep quality was rated poorly (62 ± 18 on a VAS scale of 0 to 100) The VAS ratings for sleep quality were higher after landing compared with ratings obtained during shuttle missions (*p* < .0001), along with3 months (*p* = .024), and 11 days (*p* = .003) before spaceflight. The VAS ratings for alertness were higher in the first 7 days after landing compared with the VAS values during shuttle missions (*p* = .001) and 11 days before spaceflight (*p* = .033). Subjective sleep quality was significantly higher in the first 7 days after landing compared with the in-flight periods (*p* < .0001) and during preparations before spaceflight (*p* = .003)
5. Flynn-Evans et al. [44] **Aim**: To determine how PVT and fatigue ratings changed during a simulated mission and to evaluate bio-mathematical predictive models	16 (6% women) 39 ± 8	Healthy volunteers recruited to simulate***astronauts’*** activities **Laboratory** (United States) Observational study with a stimulated 45-day space mission Rest schedules allowed for 5 hrs of sleep during weekdays (0200–0700) and 8 hrs of sleep on weekends (1100–0700; 2 nights)	**Fatigue (subjective):** Samn-Perelli scale **Performance (objective):** PVT *No objective measures of sleep were examined*	*Circadian rhythms* *were not examined*	*Sleep duration was not reported* Samn-Perelli scale scores did not change significantly during the study, but the fatigue scores were significantly greater after 5 hrs of sleep versus 8 hrs of sleep (*p* < .001). Subjects’ performance on the PVT worsened from the beginning to the end of workdays (*p* = .048) when the data were averaged across all study days. Compared with weekends (after an 8-h sleep opportunity) the following PVT measures were slower on weekdays (5-h sleep opportunity): RT, fastest 10% RT, slowest 10% RT, and response speed. In addition, the average number of lapses was higher (*p* < .01 for all comparisons) Bio-mathematical models were used to predict PVT performance based on Samn-Perelli fatigue ratings. Four models were able to predict relative changes in performance according to the day of the mission and by session of the day but were less accurate at capturing differences in PVT performance in the days following the 5 hrs of sleep condition
6. Flynn-Evans et al. [45] **Aim**: To determine how work start time affected 6-sulfatoxymelatonin levels, sleep duration and timing, fatigue, and performance	44 (9% women) 31 ± 7	***Airline pilots*** (short-haul) **Field** (United States) Baseline duty time was mid-morning (start time 1030); compared with early (0530 start), high-workload mid-day (1400) and late (1630)	**Sleep (objective):** Actigraphy **Sleep (subjective):** Diaries **Circadian (objective):** Urinary 6-sulfatoxymelatonin levels **Circadian (subjective):** Morningness-Eveningness Questionnaire **Fatigue (subjective):** Samn-Perelli scale **Performance (objective):** PVT	The mean circadian phase of the 6-sulfatoxymelatonin levels was shifted earlier when pilots had early take-offs (compared with the mid-morning take-offs during the baseline period; *p* = .004). No phase shift was associated with mid-day and late flights compared with baseline (*p* = .67and .30, respectively)	Pilots slept 5.7 ± 0.7 hrs on the night before an early-take-off (compared with 6.8 ± 0.9 hrs before later flights, *p* < .01). The WASO was longer before earlier flights than mid-morning flights (54 ± 37 versus 45 ± 30 min, *p* < .05) When pilots slept during different times of day, they did not rate the quality of their sleep differently. Sleep diary data revealed that pilots overestimated their total sleep duration (as compared with actigraphy-derived sleep measures) Mean PVT RT was significantly slower for the early, mid-day and late start times compared with baseline (i.e. mid-morning start time). Comparisons of mean PVT lapses, RT, and response speed were not significantly different between mid-morning take-offs and pilots’ off-duty days
7. Flynn-Evans et al. [46] **Aim**: To define circadian misalignment in space and determine how sleep was associated with mission-critical events	21 (29% women) 47 ± 4	***Astronauts*** **Field** (Space) *Work and sleep times were not reported*	**Sleep (objective)**: Actigraphy **Sleep (subjective):** Diaries (e.g. number of awakenings were documented), VAS rating of sleep quality (ranging from 0 to 100) **Fatigue (subjective):** VAS ratings of alertness (ranging from 0 to 100) **Circadian (objective):** Temperature (Circadian Performance Simulation Software) *No objective measures of performance were examined*	The circadian temperature rhythm did not align with 13% of astronauts’ sleep episodes pre-flight. In-flight, 19% of the sleep episodes were not optimally aligned with the circadian temperature rhythm Pre-flight, astronauts slept significantly longer when their circadian temperature rhythm was aligned with their sleep timing (6.0 ± 1.2 hrs), compared with when it was misaligned (5.5 ± 1.8 hrs; *p* = .002). In-flight, sleep was also significantly longer when aligned with the temperature circadian rhythm (6.4 ± 1.2 hrs) compared with circadian misaligned sleep (5.4 ± 1.4 hrs; *p* < .0001) Sleep quality ratings were higher (66.8 ± 17.7) when the circadian temperature rhythms were aligned with astronauts’ sleep timing (misaligned = 60.2 ± 21.1; *p* = .01). Alertness ratings, however, were not different depending on whether sleep timing was aligned with the circadian rhythm (*p* = .130)	Sleep durations were 5.9 ± 0.9 hrs pre-flight and 6.1 ± 0.7 hrs in-flight (according to actigraphy) According to sleep diaries, sleep durations did not differ between the pre-flight (6.3 ± 0.8 hrs) and in-flight periods (6.5 ± 0.7 hrs). Sleep quality measures were also similar (pre-flight, 62.3 ± 15.6; in-flight, 66.5 ± 13.4). Astronauts‘ alertness rating did not differ pre-flight versus in-flight (56.0 ± 19.5 pre-flight and 57.7 ± 18.7 during space flight
8. Gander et al. [47] **Aim**: To examine the effect of multiple trans-meridian flights and in-flight sleep duration on performance	39 (0% women) 55	***Airline pilots*** (long-haul) **Field** (United States [East Coast or Hawaii] to Japan) Day and night duty periods analyzed. Flight from the East Coast departed at 1400 hrs or 0230 hrs, and they arrived in Hawaii at 0345 or 1500 hrs. Flight departed Hawaii at 1700 hrs and arrived in Japan at 1700 hrs. Flights departed Japan at 1700 or 0800 hrs and arrived in the United States at 0330 hrs or 1530 hrs	**Sleep (objective)**: Actigraphy **Sleep (subjective):** Diaries **Fatigue (subjective):** KSS; Samn-Perelli scale **Performance (objective):** PVT	*Circadian rhythms* *were not examined*	In-flight sleep duration was ~3 hrs on flights travelling from the East coast to Japan; the relief crew for landing obtained 2.3 hrs of in-flight sleep After a long-haul flight leg, pilots obtained significantly more sleep on post-trip day one compared to baseline (estimated mean sleep extension = 49 min, *p* = .008). From post-trip day two onwards, total sleep time per 24 hrs was not significantly different from baseline Total sleep time (in the prior 24 hrs) predicted KSS scores at TOD. In flights from Japan to the East Coast (United States of America), total sleep time in the prior 24 hrs was associated with mean RT and slowest 10% RT (at TOD). In the Japan to East Coast flight pattern, there was a main effect of total time awake on the slowest 10% RT
9. Gander et al. [48] **Aim**: To combine sleep/wake history data from four studies of long-range and ultra-long-range flights to test association with fatigue and performance	237 (% women not reported) 43 (median, range 27–63)	***Airline pilots*** (long-haul) **Field** (around the world) Day and night duty periods analyzed. Departure time was categorized in 4-hr bins: 0200–0559, 1000–1359, 1400–1759, 1800–2159, and 2200–0159	**Sleep (objective)**: Actigraphy **Sleep (subjective)**: Diaries **Fatigue (subjective)**: KSS, Samn-Perelli scale **Performance**: PVT		The median sleep duration prior to duty was 7.0 hrs (range 2.2–13.8 hrs). Median total sleep time in-flight was 3.7 hrs (range 0.2–6.3 hrs). Median total sleep time in the 24 hrs prior to TOD was 4.8 hrs (range 0.2–11.5 hrs) For every 1-hr increase in sleep duration, the pre-flight KSS ratings and Samn-Perelli fatigue ratings decreased significantly. The duration of time awake at duty start was not associated with KSS or Samn-Perelli scores. Total sleep time in the 24 hrs before duty was associated with an increase in PVT response speed. The duration of time awake (at duty start) was not associated with PVT response speed At TOD, time awake was associated with an increase in KSS and Samn-Perelli scores. Total sleep time (in the 24 hrs prior) was not associated with TOD KSS or Samn-Perelli scores. Total in-flight sleep was associated with a decrease in KSS and Samn-Perelli scores (TOD). Total sleep time in the prior 24 hrs was not associated with the slowest 10% RT on the PVT (TOD). The median KSS rating (pre-flight) was 3 (range 1–8) and at TOD was 3 (range 1–9). Median Samn Perelli rating (pre-flight) was 2 (range 1–6) and at TOD was 3 (range 1–7)
					Pilots’ KSS scores (pre-flight) were lower when flights departed between 1400 and 1759 hrs (compared with departures occurring between 2200 and 0159 hrs, *p* = .003, or between 0200 and 0559 hrs, *p* = .001). The Samn-Perelli fatigue scores (pre-flight) were lower for departures between 1400 and 1759 (compared with departures occurring between 2200 and 0159 hrs, *p* = .0003, or 0200 and 0559 hrs *p* = .0004). The KSS and Samn-Perelli scores were higher (at TOD) when flights arrived between 0600 and 0959 hrs compared with later landing times Pre-flight PVT response speeds and flight departure time post hoc analyses were not significant after correcting for multiple comparisons The mean PVT response speed and the slowest 10% of PVT responses were significantly faster at TOD for flights arriving between 1400–1759 hrs versus flights arriving earlier in the day Median PVT response speed pre-flight was 4.27 (range 1.81–5.49) and at TOD was 4.00 (range 2.11–5.41). Median slowest 10% of PVT response speed pre-flight was 2.82 (range 0.37–4.45) and at TOD was 2.55 (range 0.38–4.48)
10. Gander et al. [49] **Aim**: To determine the effects of departure and arrival times on fatigue and performance using combined analyses of data about long-range and ultra-long-range flights	237 (% women not reported) 43 (median; range 27–63)	***Airline pilots*** (long-haul) **Field** (Around the world) Flight departure times and arrival times were categorized in 4-hr bins according to the following clock hours: 0200–0559, 0600–0959, 1000–1359, 1400–1759, 1800–2159, 2200–0159	**Sleep (objective)**: Actigraphy **Sleep (subjective)**: Diaries **Fatigue (subjective):** KSS; Samn-Perelli scale **Performance**: PVT	*Circadian rhythms* *were not examined*	*Sleep duration was not reported* Pilots obtained more in-flight sleep on flights departing 1800–2159 and 2200–0159 versus 0200–0559 (*p* = .0036; *p* = .0169), 1000–1359 (*p* = .0001; *p* = .0010), or 1400–1759 (*p* = .0001; *p* = .0001) Pilots were less fatigued before flights departing 1400–1759 and 1800–2159 versus 2200–0159 (*p* = .0021; *p* = .0315), or 0200–0559 (*p* = .0013; *p* = .0270). At TOD, pilots had higher KSS and Samn-Perelli ratings on flights arriving 0600–0959 versus 1000–1359 (*p* = .0005; *p* =.0347) Flight departure time impacted KSS and Samn-Perelli ratings (*p* = .0002 and *p* < .0001). Pilots were less sleepy before flights departing 1400–1759 versus 2200–0159 (*p* = .0204) or 0200–0559 (*p* = .0032). Flight departure time did not impact pre-flight PVT metrics. At TOD, mean RT was slower on flights arriving 0200-0959 versus arriving 1400–1759 (0200–0559, *p* = .003; 0600–0959, *p* = .004). Mean RT was also significantly slower at TOD on flights arriving 0600–0959 than on flights arriving 1400–1759 (*p* = .004)
11. Ganesan et al. [50] **Aim**: To examine the impact of one versus two consecutive nights of work on fatigue and performance	29 (28% women) 33.4 ± 10.1	***Truck drivers*** **Field** (Australia) Working 12-hr night-shifts in a coal mine in Australia (data were collected over 2 consecutive night-shifts) All participants worked day shifts followed by a minimum of 2 days off work	**Sleep (objective)**: Actigraphy **Sleep (subjective)**: Diaries; PSQI **Fatigue (subjective):** KSS; Epworth Sleepiness Scale **Performance:** PVT; driving	*Circadian rhythms* *were not examined*	Sleep duration differed before the first and second work nights (9.1 ± 1.5 versus 5.4 ± 1.2, *p* < .01). There were increased KSS scores at the end of both shifts (effect of time; *p* = .001) PVT lapses and the slowest 10% of RT were similar at the start and end of the first night of work. Greater impairments in performance occurred at the end of the second night of work (versus night one; *p* < .05). There was also a significant shift in the time interaction observed on drive violations (*p* = .05). Total sleep time in the 48 hrs prior to the start of shifts significantly impacted performance during night shift-work (*p* = .001)
12. Gregory et al. [51] **Aim**: To measure the impact of sleep duration and quality (from in-flight rest breaks) on fatigue	500 (% women not reported) *Age not reported*	***Airline pilots*** (short-haul) **Field** (United States) Day and night duty periods analyzed. Time of day during in-flight rest breaks categorized into 4-hr bins: 0200–0600, 0600–1000, 1000–1400, 1400–1800, 1800–2200, 2200–0200 hrs	**Sleep (subjective):** Self-reported in-flight sleep and quality (1 = very good; 5 = very poor) and duration **Fatigue (subjective):** KSS, Samn-Perelli scale *No objective measures of sleep or performance were examined*	*Circadian rhythms* *were not examined*	Only in-flight sleep duration was reported (1.5 ± 0.7 hrs from clock time 0600–1000, 1.2 ± 0.7 hrs from 1400–1800, and 1.4 ± 0.7 hrs from 2200 to 0600) Samn-Perelli ratings (at break start) during the night-time (from 0200–0600 hrs) were higher versus the afternoon and early evening. Samn-Perelli ratings during the early evening (1800–2200 hrs) were lower than earlier (0600–1400 hrs) and later (2200-0200 hrs). KSS ratings at break start during the night-time and early-morning (2200–1000 hrs) were higher than those during the afternoon and early evening (1400–2200hrs). Samn-Perelli ratings at TOD during the night-time (0200–0600 hrs) were significantly higher than those obtained during the mid-day (1000–1400 hrs). The KSS ratings at TOD did not differ significantly by time
13. Gregory et al. [52] **Aim:** To examine scheduling practices (over 1 year) and test a fatigue prediction model	61 (% women not report) ≥ 30	**Airline pilots** (maritime) **Field** (United States) Work periods lasted 7.6 hrs on average (pilots had ≤ 4 ship assignments per work period). Work weeks were 35 hrs on average (usually for 3 consecutive days). Night work typically started at 0200 hrs	**Fatigue:** The Sleep, Activity, Fatigue, and Task Effectiveness-Fatigue Avoidance Scheduling Tool *No objective measures of sleep, subjective measure of fatigue, or objective measures of performance were examined*	*Circadian rhythms* *were not examined*	Over half of the work periods were assigned high predicted fatigue scores. Higher predicted fatigue was associated with duty starting between 0000 and 0400 hrs. Variability in start times and busy assignments were also associated with higher predicted fatigue scores
14. Honn et al. [53] **Aim**: To examine the impact of multiple flights during the day versus one flight on fatigue and performance	24 (8% women) 33 (range 24–49)	***Airline pilots*** **Laboratory** (flight simulator) Duty periods lasted from 0515 to 1415 hrs. Pilots flew in the stimulator for 2 consecutive days. A randomized cross-over design was used so that pilots had multiple flights one day and flow on flights the other day	**Sleep (objective):** Actigraphy **Sleep (subjective):** Diary **Fatigue (objective):** KSS; Samn-Perelli Fatigue Scale **Performance:** PVT	*Circadian rhythms* *were not examined*	The night before the first simulated flight, pilots slept 5.6 ± 1.1 hrs. Before the second day, they slept 6.4 ± 1.2 hrs There were significant reductions in Samn-Perelli (by 0.5 ± 0.1) and KSS scores (by 0.4 ± 0.1) for every additional hour of sleep pilots obtained before flying. Prior sleep duration was negatively associated with scores on the Samn-Perelli scale (*p* < .001) and KSS (*p* < .001). Samn-Perelli and KSS scores were highly correlated over subjects and across test bouts (*r* = 0.63, *p* < .001). PVT lapses and mean RT were highly correlated over subjects and across test bouts (*r* = 0.67, *p* < .001). There was an increase in Samn-Perelli and KSS scores throughout the day over the test bouts regardless of the multi-flight or single segment condition. The number of PVT false starts was negatively associated with the prior night’s sleep duration (*p* < .001)
15. Jones e al. [54] **Aim**: To evaluate the impact of spaceflight on sleep–wake activities, workload, stress, fatigue, and performance	24 (21% women) 48 ± 5	***Astronauts*** **Field** (Space) Astronauts on-duty other than scheduled sleep time (2130–0600)	**Sleep (subjective):** Dairy logging daily sleep–wake schedule; VAS for sleep quality (11-point scale) **Fatigue (subjective):** VAS (11-point scale) for workload, stress, mental fatigue, physical exhaustion, tiredness, and sleepiness **Performance (objective):** Reaction Self-Test using the PVT-B *No objective measures of sleep were examined*	*Circadian rhythms* *were not examined*	Astronauts reported an average nightly sleep duration of 6.5 ± 1.4 hrs in-flight in space and only slept the NASA scheduled 8.5-hr sleep opportunity on 6% of nights Sleep time ≤6 hrs/night occurred 40% of the time on weekdays versus 23% of the time on weekends. Astronauts did not report changes in their WASO or changes in sleep efficiency while on the ISS Sleep durations <7 hrs/night were associated with poorer ratings of sleep quality, and >9 hrs of sleep was associated with more optimal ratings of sleep quality. Their VAS ratings of sleepiness, tiredness, mental fatigue were higher after ≤4, 4–5, and 5–6 hrs of sleep (versus 7–8 hrs) Sleep duration was positively associated with the mean RT on the PVT-B. Total sleep time >9 hrs was associated with faster RT relative to all groups with total sleep time ≤7 hrs
16. Koller et al. [55] **Aim**: To characterize slow-wave density, spectral band-power, duration, and slope using EEG sleep data that were collected before, during, and after two Space Shuttle missions	4 *Sex and age not reported*	**Astronauts** **Field** (Space) *Sleep and work schedules were not reported*	**Sleep (objective):** EEG *No objective measures of fatigue or performance were examined*	*Circadian rhythms* *were not examined*	*Sleep duration was not reported* The sleep period time was 42.0 ± 7.9 min shorter in-flight compared to pre-flight (*p* = .001) and 34.3 ± 9.9 min shorter compared to post-flight (*p* = .013). Total sleep time was significantly reduced by 48.2 ± 16.0 min in-flight compared to pre-flight (*p* = .029). The duration of sleep stages and WASO did not differ pre-flight versus in-flight versus post-flight. Slow spindle frequency was significantly affected by pre-flight versus in-flight versus post-flight condition (*p* < .01). There was a slow spindle shift towards higher frequencies by 0.17 ± 0.03 Hz in-flight compared to pre-flight (*p* < .001). The spindle frequency increased by 0.27 ± 0.06 Hz in-flight compared to pre-flight (*p* < .001). Slow-wave amplitude decreased by 2.53 ± 0.70 μV in-flight compared to pre-flight (*p* = .011). Slow-wave duration or band-power were not affected by flight condition (*p* = .54)
17. Mollicone et al. [56] **Aim**: To predict fatigue from sleep/wake patterns using a bio-mathematical fatigue model examine relationships between predicted fatigue levels and hard-braking events	106 (6% women) 45 ± 11	***Truck drivers*** **Laboratory** (United States) *Schedules were not reported*	**Sleep (objective)**: Actigraphy **Fatigue (subjective):** KSS **Performance (objective):** PVT; hard-braking events	*Circadian rhythms* *were not examined*	*Sleep duration was not reported* Of the total hrs truck drivers were driving, 50% were associated with predicted fatigue scores ≥5.9 (median); 5% were associated with predicted fatigue scores ≥12.8, 1% were associated with predicted fatigue scores >16.5 The frequency of hard-braking events increased as predicted fatigue levels worsened, after controlling for time of day (*p* = .018). Of the hard-breaking events, 58% were associated with predicted fatigue (derived from sleep/wake by actigraphy) ≥5.9; each 1-unit increment on the fatigue scale (equivalent to 1 lapse on the PVT) increased the frequency of hard-braking events by 7.8%
18. Moore et al. [57] **Aim**: To determine the impact of long-duration spaceflight and microgravity on post-landing motor vehicle operator proficiency	8. Tested before and after 142–200 days on the ISS (0% women) 48 ± 7 12 Controls (ground-based healthy subjects; 0% women) 39 ± 10 12 (sleep-restriction group) 40 ± 11 (44% women)	***Astronauts*** **Laboratory** (United States)	**Fatigue (subjective):** Stanford Sleepiness Scale **Performance (objective):** Cognitive/sensorimotor test battery *No objective measures of sleep were examined*	*Circadian rhythms were not examined*	Upon returning from the ISS, astronauts demonstrated significant deficits in manual dexterity, dual-tasking, motion perception, and the ability to operate a motor vehicle. Astronauts post-flight motor functions recovered (returned to baseline) 4 days after landing
19. Morris et al. [58] **Aim**: To determine relationships among pilot’s perceptions of fatigue, circadian preferences, fatigue mitigation strategies, and performance	21 (14% women) 29 ± 2	***Airline pilots*** (Cargo; Air Force) **Field** (United States) *Schedules were not reported*	**Sleep (objective):** Actigraphy (but data were not reported) **Fatigue (subjective):** Fatigue perceptions (five items ranging from 1 = strongly agree and 5 = strongly disagree); five fatigue mitigation strategies (responding never, sometimes, half of the time, most of the time, and always) **Performance (objective):** PVT (but data were not reported)	*Circadian rhythms* *were not examined*	*Sleep duration was not reported* Pilot perceptions of fatigue as a serious safety of flight concern was positively associated with personal concerns of fatigue (*r* = 0.67, *p* < .01), and the perception that changes are needed to address pilot fatigue (*r* = 0.55, *p* < . 05). The perception that changes are needed to address pilot fatigue was positively associated with personal concerns of fatigue (*r* = 0.57, *p* < .01), and pilots reported feeling pressured to continue a mission despite fatigue (*r* = 0.60, *p* < .01) In this sample, 5% of pilots a had a “moderate evening” chronotype, 29% were “intermediate evening”, 33% were “intermediate morning”, and 33% were “moderate morning”. In addition, 67% of pilots responded, “strongly agree” to the question: “Fatigue is a serious safety of flight concern for the air mobility community”. 38% of pilots responded, “strongly agree” and 38% of pilots responded “agree” to the question: “I have personally felt concerned about my fatigue level with respect to safety of flight”
20. Nasrini et al. [59] **Aim**: To evaluate fatigue and performance after sleep-restriction	32 (53% women) 36 ± 8	***Astronauts*** **Laboratory** (spaceflight mission simulator) Day and night duty periods analyzed. Astronauts on-duty other than scheduled sleep time (2300–0700). In campaign 1, participants underwent one-night of sleep-restriction (scheduled sleep time 0300–0700). In campaign 2 participants underwent one-night of sleep deprivation (no scheduled sleep time)	**Sleep (subjective)**: Time in bed; Perceived sleep quality (Campaign 2 only) **Fatigue (subjective)**: Perceived alertness; Perceived workload, sleepiness, happiness, sickness, physical exhaustion, mental fatigue, stress, depression, boredom, loneliness, monotony, and crewmember conflicts (campaign 2 only) **Performance (objective):** Cognition test battery and PVT *No objective measures of sleep were examined*	*Circadian rhythms were not examined*	*Sleep duration was not reported* Over time (excluding sleep deprivation days), there were decreasing ratings of poor sleep quality in campaign 2. In general, subjects reported high sleep quality. Subjects were more likely to report tiredness during sleep deprivation in both campaigns. In campaign 2 there was a significant effect of sleep deprivation on subjects reporting increased workload, sleepiness, physical exhaustion, and mental fatigue Accuracy on the PVT increased significantly over time in mission (excluding sleep deprivation days). Reduced sleep impaired PVT response speed and accuracy was lower (*p* < .001)
21. Powell et al. [60] **Aim**: To evaluate a bio-mathematical model to predict fatigue	324 (53% female) *Age not reported*	***Airline pilots*** (long and short-haul) **Field** (New Zealand and Australia) Data analyzed from 11 studies across both day and night duty periods	**Sleep (subjective):** Diary (documenting in-flight sleep periods and sleeping locations) **Fatigue (objective):** Pilot Alertness Test **Fatigue (subjective):** KSS; Samn-Perelli fatigue scale; VAS for fatigue and drowsiness; System for Aircrew Fatigue Evaluation (SAFE) model for predicting fatigue levels from in-flight sleep and duty information **Performance (objective):** Pilot Alertness Test *No objective measures of sleep were examined*	*Circadian rhythms* *were not examined*	*Sleep duration was not reported* Predicted fatigue values from the SAFE model were positively associated with VAS scores (*r* = 0.85). Predicted fatigue values were positively associated with RT on the Pilot Alertness Test (*r* = 0.57). The SAFE model overestimated fatigue during the morning and underestimated fatigue during the evening. A similar trend was noted for over- and under-estimation of the RT during the morning and evening (not statistically significant). Subjective fatigue (VAS) was positively associated with the RT determined by the Pilot Alertness Test (*r* = 0.74)
22. Sallinen et al. [61] **Aim**: To examine aircrew fatigue during night duty shifts	392 (24% women) Mean age not reported	***Airline pilots*** (long and short-haul) **Field** (European Union) Day and night duty periods were analyzed. The analysis focused on high-risk schedules: night work >10 hrs and disruptive schedules (i.e. early-starts, late finishes, and nights)	**Sleep (objective):** Actigraphy **Fatigue (subjective):** KSS; Samn-Perelli scale **Performance (objective):** PVT-B	*Circadian rhythms* *were not examined*	On-duty napping occurred during long night duty periods (pilots: 31%, cabin crew: 20%) and short night duty (pilots: 11%, cabin crew: 8%) of employees. Prior sleep duration was a main predictor of high fatigue levels. The probability of high fatigue (KSS ≥7 at TOD) was 0.41 and 0.32 during long (>10 hr) and short (<10 hr) night duty periods. Start time was a significant predictor of fatigue levels. When the duty period occurred between 0200 and 0559 hrs, higher fatigue levels were more likely
23. Sallinen et al. [62] **Aim**: To determine levels and predictors of fatigue at TOD and determine the in-flight sleep/wake ratio according to the ‘window of circadian low’	519 (18%women) 38 ± 9	***Airline pilots*** (long and short-haul) **Field** (European Union) Day and night duty periods analyzed. Analysis focused on high-risk schedules: >10 on-duty hrs at night and schedules that are considered disruptive of circadian rhythm (early-starts = duty period start time between 0500 and 0659 hrs; late finishes = duty period end time between 2300 and 0159 hrs; FDPs during the window of circadian low between 0200 and 0459 hrs)	**Sleep (objective):** Actigraphy **Sleep (subjective):** Diary; Morningness-Eveningness Questionnaire **Fatigue (subjective):** KSS; Samn-Perelli scale **Performance:**PVT (pilots only)	*Circadian rhythms were not examined*	On-duty napping occurred in 5% of early-starts (pilots: 8%, cabin crew: 1%), 11% of late finishes (pilots: 12%, cabin crew: 8%), and 15% of night FDPs (pilots: 16%, cabin crew: 13%) High KSS scores at TOD were more likely during night-shifts (both long and short flights). Point estimates for high KSS scores were higher for both types of shifts with consecutive late finishes or night-shifts. Start time, end time, number of time zones crossed, and number of shifts during the ‘window of circadian low’ were significant predictors of high KSS scores at TOD The mean KSS score at TOD was 5.4 for both long and short nights. The odds ratio for high fatigue (KSS) at TOD was 2.0 during early-starts (*p* = .026), 3.8 during late finishes (*p* < .001), and 3.0 during nights (*p* < .001; compared with daytime as a reference). Longer time awake at TOD was associated with increased odds of reporting high KSS scores (*p* < .001). More hrs of sleep in the previous 24 hrs were associated with lower odds of reporting high KSS scores at TOD (*p* < .001)
24. Sparrow et al. [63] **Aim**: To determine how restart breaks affected sleep, fatigue, and performance in daytime and night-time drivers	106 (6% women) 45 ± 11	***Truck drivers*** **Field** (United States) Day and night duty periods analyzed. Time of day (hr) was used in statistic models	**Sleep (objective):** Actigraphy **Fatigue (subjective)**: KSS **Performance:** PVT, driving activity	*Circadian rhythms were not examined*	Before restart breaks, drivers slept a mean of 6.0 ± 0.2 hrs per 24 hrs before the one-night restart condition, and 6.2 ± 0.1 hrs in the > one-night restart group. The one-night restart group slept an average of 8.8 ± 0.3 hrs per 24 hrs during the restart break and the > one-night restart group slept an average of 8.9 ± 0.2 hrs The KSS scores were higher in the one-night restart condition. The 24-hr patterns of lane deviation in the two conditions were consistent with the 24-hr patterns observed for the PVT-B. On PVT-B, drivers exhibited 2.0 ± 0.3 lapses of attention during duty cycles preceded by the one-night restart condition, and 1.7 ± 0.3 lapses of attention per PVT-B assessment during duty cycles preceded by the > one-night restart condition (*p* = .015) There was a significant interaction of condition (one-night versus > one-night restart group) and hour of the day for sleep obtained during both duty time and the restart break. The one-night restart group obtained more of their sleep during daytime hrs, while the > one-night restart group obtained more sleep at night In the one-night restart group, KSS scales were significantly higher during daytime periods. There was a significant effect of both condition and time of day on PVT lapses. There was a significant interaction of condition by hour in the number of lane deviations. Lane deviations in the one-night restart group were higher during 0400–0500, 0800–0900, and 1500–1600 hrs. Lane deviations were higher in the >1-night restart group during 1900–2000 hrs
25. van den Berg et al. [64] Aim: To determine how in-flight sleep and time of day affect fatigue	298 *Sex and age not reported*	***Airline pilots*** (long-haul) **Field** (New Zealand) Day and night duty periods analyzed. Time of day during in-flight rest breaks and arrival times categorized into 4-hr bins: 0200–0559, 0600–959, 1000–1359, 1400–1759, 1800–2159, 2200–0159	**Sleep (subjective):** Diary; sleep quality on 7-point scale where 1 = extremely good and 7 = extremely poor **Fatigue (subjective):** KSS; Samn-Perelli scale, Crew Status Check *No objective measures of sleep or performance were examined*	*Circadian rhythms were not examined*	*Sleep duration was not reported* Fewer pilots attempted to nap from 1800 to 2159 compared with 2200–0159 and 0200–0559. Pilots obtained more sleep during breaks starting 0200–0559 versus breaks starting 0600–0959 (*p* = .001), 1000–1359 (*p* = .004), and 1800–2159 (*p* < .0001). Sleep quality was rated as significantly better for breaks starting 0200–0559 than for breaks starting 1800–2159 (*p* = .001) and 2200–0159 (*p* = .032) Pilots reported higher KSS scores (at TOD) on flights arriving 0200–0559 than on flights arriving 1000–1359 (*p* < .0001), 1400–1759 (*p* < .0001), and 2200–0159 (*p* < .0001). Pilots also reported higher KSS scores at TOD on flights arriving 0600–0959 than on flights arriving between 1000–1359 (*p* < .0001), 1400–1759 (*p* < .0001), and 2200–0159 (*p* < .0001) With every 1-hr increase in sleep duration in-flight, KSS sleepiness ratings decreased by 0.6 points and Samn-Perelli fatigue ratings decreased by 0.4 points. Most pilots surveyed attempted sleep during their scheduled rest break. Samn-Perelli fatigue ratings had the same relationships with arrival times as KSS scores at TOD
26. Vejvoda et al. [65] **Aim**: To compare fatigue levels upon landing for late and early flights	40 (0% female) 32 ± 6	***Airline pilots*** (short-haul) **Field** (Germany) Early starting duty periods (0500–0659) were compared to late-finishing duty periods (0000–0159)	**Sleep (objective):** Actigraphy **Sleep (subjective):** Diaries **Fatigue (subjective):** KSS; Samn-Perelli scale; Crew Status Check The NASA Task Load index was used to measure workload complexity *No objective measures of performance were examined*	*Circadian rhythms were not examined*	The duration of the preceding night sleep time (which averaged 6.5 hrs in early starters and 7.6 hrs in late finishers, respectively) had no significant effect on fatigue (Samn-Perelli) at end of shift Pilots were significantly more fatigued when they started working in the afternoon or evening, compared with the morning (Samn-Perelli scale scores obtained at the end of duty) A comparison of early-starts and late-landings showed that pilots’ fatigue was higher for the latter condition (*p* < .001), reaching moderate to severe levels, even though the prior sleep period time was longer by 1.1 hrs (*p* < .001) and duty duration was shorter by 0.7 hrs (8.6 versus 9.3 hrs, *p* = .047) Fatigue and sleepiness increased in the late evening hrs, which were paralleled by longer time awake. Fatigue reached critical values after 2200 hrs. Fatigue was lower in the morning despite shorter previous night sleep period time. Time awake at start of shift was a main predictor Samn-Perelli scale scores (at shift end).Sleep period time before a shift had no significant effect on fatigue
27. Wong et al. [66] **Aim**: To determine how 28-hrs of sleep deprivation affected ROBoT metrics(e.g.% of successful captures, alignment-reversal score, and efficiency to capture)	9 (44%women) 32 ± 10	Healthy volunteers recruited to simulate***astronauts’*** activities **Laboratory** (NASA; spaceflight mission simulator for 28-hrs) Participants were studied during the day and night	**Sleep (objective):** Actigraphy (before sleep deprivation) **Sleep (subjective):** PSQI (global PSQI scores < 5 were required for study participation) **Fatigue (subjective):** Fatigue Severity Index (scores < 36 were required for study participation); KSS **Circadian Preference (subjective):** Morningness-Eveningness Questionnaire (scores < 58 or > 42 were required for study participation) **Performance (objective):** ROBoT; PVT	*Circadian rhythms were not examined*	Before the sleep deprivation period, subjects obtained 8.1 ± 0.5 hrs of sleep (PSQI global score = 2.8 ± 1.6) Subjects demonstrated improved performance (learning effects) over time despite sleep loss. Participants improved from the second to the last training session in the alignment-reversal score (*p* < .001), efficiency to capture (*p* = .004), and percentage of successful captures (*p* = .01). When skills were separated by difficulty, there was an improvement in the medium-easy and hard trials with increased time awake, but not other levels of difficulty. There was a significant change in several components of PVT with increased time awake including: decreased mean 1/RT (*p* < .001), decrease in slowest 10% RT (*p* < .001), increase in the fastest 10% RT (*p* < .001), and an increase in number of lapses (*p* < .001). No ROBoT performance metrics had significant correlations with PVT scores. Subjects’ KSS scores were correlated with the % of successful captures (*r* = 0.24, *p* < .05) but not with any other ROBoT metrics
28. Zaslona et al. [67] **Aim**: To qualitatively analyze pilots’ experiences with in-flight sleep and their efforts to mitigate fatigue	123 (% women not reported) *Age not reported*	***Airline pilots*** (long-haul) **Field** (New Zealand) Data from pilots working long-range and ultra-long-range flights across day and night duty periods	**Sleep (subjective):** Diaries **Fatigue (subjective):** Open-ended questions about in-flight fatigue mitigation strategies *No objective measures of sleep or performance were examined*	*Circadian rhythms were not examined*	*Sleep duration was not reported* The designs/locations of the aircrafts’ rest facilities affected pilots’ perceptions of sleep quality, fatigue, and alertness. Uncertainty about the scheduling of breaks/naps disrupted pilots’ in-flight rest, especially for junior pilots. Pilots experienced enhanced sleep quality (and improved alertness) when the timing of their in-flight naps were aligned with their perceived circadian rhythms. Pilots attempted to adjust the timing of their sleep when they knew their flight schedules in advance

Data are mean ± standard deviation (unless noted otherwise).

EEG, Electroencephalogram; ISS, International Space Station; KSS, Karolinska Sleepiness Scale (scores range from 1 [extremely alert] to 9 [extremely sleepy]); MEQ, Morningness-Eveningness Questionnaire (scores 70–86 [morning type], 59–69 [moderately-morning], 42–58 [neither type], 31–41 [moderately evening-type]), 16–30 [evening-type]); PSQI, Pittsburg Sleep Quality Index (scores range 0–21 [scores >5 indicate poorer sleep quality]); PVT, psychomotor vigilance task; ROBoT, robotics on-board trainer; RT, reaction time; TOD, top of descent; VAS: Visual Analog Scale.

## Results

The research about pilots [40–42, 45, 47–49, 51–53, 58, 60–62, 64, 65, 67], truck drivers [50, 56, 63], and astronauts [43, 44, 46, 54, 55, 57, 59, 66] included laboratory studies (e.g. conducted in stimulators) [43, 44, 53, 56, 57, 59, 66] and field investigations (where data acquisition occurred during normal occupational activities) [40–42, 45–52, 54, 55, 58, 60–65, 67]. Most pilots were employed by commercial airlines (to transport passengers) [40–42, 45, 47–49, 51, 53, 60–62, 64, 65, 67] with the exception of studies about maritime piloting (to guide ships) [52] and military transport operations [58]. Two papers about truck drivers utilized the same group of drivers (who had irregular start times) [56, 63]; another sample of truck drivers worked rotating day- and night-shifts with predictable start times [50]. Data were also provided by astronauts assigned to the International Space Station (ISS) [43, 46, 54, 57, 59] and Space Transportation System [43] as well as healthy volunteers who participated in simulated missions [44, 57, 59, 66]. The key findings across studies were organized to reflect the following themes: (1) sleep characteristics; (2) fatigue during work, (3) schedules and circadian rhythms; and (4) variables affecting performance.

### Sleep characteristics

Sleep was measured objectively in space [43, 46, 54, 55] and on Earth [41–43, 45–50, 53, 56, 61–63, 65, 66]. In addition to actigraphy [41–43, 45–50, 53, 54, 56, 61–63, 65, 66], subjects completed sleep diaries [43, 45–50, 53, 54, 60, 62, 64, 65, 67], and questionnaires, such as the Pittsburgh Sleep Quantity Index (PSQI) [40, 42, 50, 66] and Athens Insomnia Scale [40]. Polysomnography was utilized in one study for a quantitative analysis of the cortical electroencephalogram (EEG) in astronauts before, during, and after a space mission [55]. Subjects’ sleeping periods were restricted by researchers in two studies to examine the effects of sleep deprivation [44, 59]. Eleven of the articles did not provide any sleep duration data [40, 44, 49, 55, 56, 58–61, 64, 67]. When sleep durations were calculated, sleeping ~6 hrs/day was common in all three occupations [43, 45, 46, 53, 54, 63, 65]. Truck drivers, for example, slept 6.2 ± 0.1 [mean ± standard error] hrs/day before driving, and they did not increase their sleep significantly when off-duty [63]. Airline pilots had mean sleep durations >7 hrs in three studies [41, 42, 53]. Pilots’ sleep durations were affected by their subsequent flight schedules [42, 45, 48, 49, 64, 65]. For example, early-morning take-offs were associated with shorter sleep durations in short-haul pilots (compared with their sleep duration before flights with later departure times) [42, 45]. On long-haul flights, pilots’ obtained more in-flight sleep when their flights departed later in the day (after 1800 hrs) compared with earlier departure times [49].

The data from astronauts illustrated their difficulties with sleeping in space. Astronauts commonly used medications to sleep, such as zolpidem [43], zaleplon [43], and melatonin [55] (the use of sleep-inducing medications was not examined in pilots or truck drivers). According to polysomnography recordings analyzed by Koller et al. [55] , astronauts had a significant reduction in daily total sleep time by 0.8 ± 0.3 hrs when they were in space, compared with their sleep on Earth. In other studies about astronauts, shorter sleep durations and poor sleep quality were associated [54, 59]; astronauts also rated the quality of their sleep in space poorly (compared with their sleep on Earth) [43]. Importantly, astronauts did not obtain >6 hrs of sleep on the nights before the more dangerous aspects of their missions, such as extravehicular activities (EVA) [43].

Koller et al. [55] hypothesized that microgravity may physiologically alter the structure of sleep in space. To test this hypothesis, four astronauts underwent polysomnography recordings. Koller et al. focused on two features of non-rapid eye movement (NREM) sleep: sleep spindles and slow-waves. Sleep spindles—bursts of oscillatory activity (9–15 Hz) lasting <2 s—have been associated with memory processing, while slow-waves indicate the depth and quality of NREM sleep. Sleep spindles can be fast (12–15 Hz) or slow (9–12 Hz) and occur during stages 2 and 3 of NREM sleep. Compared with sleep on Earth, the astronauts’ sleep in space was characterized by lower slow-wave amplitude (decreased by 2.5 ± 0.7 µV in-flight versus pre-flight, *p* = .01). They also demonstrated a higher fast spindle density (increased by 1.8 ± 0.5 spindles/min in-flight versus pre-flight, *p* < .001) and a shift toward higher frequencies in the slow spindles (increased by 0.2 ± 0.03 Hz in-flight versus pre-flight, which returned to baseline when astronauts returned to Earth). The following parameters were examined but did not differ significantly in the space/Earth comparisons: the duration of sleep stages, wake after sleep onset (WASO), fast spindle amplitude, slow spindle density, and slow spindle band-power. Collectively, the findings indicated that the depth of sleep may be reduced in space, and Koller et al. [55] also suggested that the sleep spindle changes could impact the ability to learn new skills, such as adapting to weightlessness in space, although this study did not demonstrate that any specific cognitive tasks were correlated with sleep microstructural changes .

### Fatigue during work

Five validated questionnaires were used to subjectively measure fatigue: the Karolinska Sleepiness Scale (KSS; scores range 0–9) [42, 47, 49–51, 53, 56, 60–66], Stanford Sleepiness Scale (scores range 1–7) [57], Epworth Sleepiness Scale (scores range 0–24) [40, 42, 50], and Fatigue Severity Scale (scores range 9–63) [40, 66], and Samn-Perelli fatigue scale (scores range 0–7) [41, 42, 44, 45, 47–49, 51, 53, 60–62, 64, 65]. Questionnaires were not administered at similar times across the 28 studies. For example, truck drivers’ fatigue questionnaires were completed three times a day [63] or at the beginning and end of their shifts [50]. Pilots’ fatigue levels were usually measured at Top of Descent (TOD, a safety-sensitive time during flight [~30 min before landing]) [47–49, 51, 61, 62, 64, 65], although pilots also completed questionnaires when they reached their cruising speed [53] or every morning and evening [42]. Maritime pilots did not complete any validated fatigue questionnaires, however, data about their work schedules were used to calculate ‘effectiveness’ scores that reflected their predicted levels of fatigue [52]. Astronauts’ questionnaires, including fatigue visual analogue scales (VAS), were typically administered immediately before or after they engaged in performance testing [44, 46, 54, 57, 59]. Some of the papers did not specify the exact times when subjects were asked to complete questionnaires or VAS ratings [40, 43, 58].

Questionnaire data revealed that truck drivers’ fatigue was affected by their work and rest schedules [50, 56, 63]. Over 12-hrs of driving, for example, truck drivers’ KSS scores increased throughout their shift (4.1 ± 1.4 to 6.3 ± 1.5 [mean ± standard deviation]; *p* < .01) [50], and truck drivers’ on-duty KSS scores were lower after they had >1 nighttime rest period (3.3 ± 0.1 [with only 1 nighttime rest period] versus 3.1 ± 0.1 [mean ± standard error], *p =* .03) [63]. For astronauts, fatigue levels differed according to whether measures were obtained pre-flight, during spaceflight, or upon returning to Earth [43, 57]. When astronauts returned from the ISS, for example, their Stanford Sleepiness Scale scores were higher than their pre-flight scores (4.0 ± 1.6 versus 2.1 ± 0.6, *p* > .05) [57]. Two studies about astronauts, however, did not find fatigue to vary significantly over time and with respect to mission timelines [46, 54].

The data from pilots illustrated how their fatigue levels were affected by numerous factors: rank/experience [40, 67], in-flight nap opportunities [67], flight durations (short-haul [≤3 hrs] or long-haul [>6 hrs] flights) [65], take-off and landing times [42, 49, 64], high workloads (e.g. multiple assignments/landings per day) [40, 52, 53], and the duration of time spent awake [48, 65]. A comparison of Captains and First Officers revealed that the more experienced pilots (Captains) reported lower Epworth Sleepiness Scale scores (7.7 ± 3.9 versus 9.7 ± 3.8, *p* < .001), and Fatigue Severity Scale scores were inversely correlated with pilots’ age (*r* = −0.79, *p* < .0001) [40]. A qualitative analysis of pilots’ perspectives about their napping opportunities on long flights supported the conclusion that experience provided them with skills for combating fatigue and staying focused—experienced pilots explained how they learned to adapt to their work conditions over time, which allowed them to use their in-flight/pre-scheduled nap periods to effectively mitigate fatigue while their co-pilots were flying [67]. Long-haul pilots reported higher levels of fatigue (Samn-Perelli scale) when they had landings in the late evening or night [65], and fatigue scores were higher (Samn-Perelli and KSS at TOD) when pilots were flying between 0200 and 0600 hrs [62]. Two studies about short-haul pilots found that they did not experience significantly different Samn-Perelli scores at TOD depending on whether they flew earlier or later in the day [42, 45], but pilots in another study had higher Samn-Perelli scale scores for early-morning versus late-morning flights (4.0 ± 0.9 versus 3.5 ± 0.8, *p* < .001) [42]. Data from the KSS (at TOD) also showed that pilots were significantly sleepier for early-morning versus nighttime landings (compared with mid-day measures) [49, 62, 64, 65].

In pilots, there were inconsistent results about the effects of prior sleep duration on KSS and Samn-Perelli fatigue scale sores—some investigators found that pilots’ prior sleep duration was significantly correlated with these fatigue measures [53, 61] while others did not find significant associations between sleep duration and fatigue [65]. For example, Honn et al. [53] studied pilots who were using a flight simulator; when they had longer sleep before their simulated flights, their KSS and Samn-Perelli fatigue scores were significantly lower (*p* < .001)—every additional hour of sleep reduced their KSS scores by 0.4 ± 0.1 units and Samn-Perelli scores by 0.5 ± 0.1 units. In addition, Gander et al. [48] found that for every additional hour that short-haul pilots had been awake, there were significant increases in Samn-Perelli and KSS scores (at TOD) by 0.1 and 0.2 points, respectively.

### Schedules and circadian rhythms

The reviewed studies involved many different types of work schedules. Unpredictable and irregular working hrs were particularly common in the transportation industry [40–42, 45, 48, 50, 52, 60–63, 65, 67]. Maritime pilots, for example, worked ‘on-call’ schedules that depended the movements of oil tankers, container ships, and cruise ships—these pilots’ start times and duty durations were highly-variable [52]. Long-haul pilots had pre-determined schedules; however, they crossed multiple time zones during their flights, which shifted their clocks by 5–12 hrs [48, 49]. Commercial airline pilots often flew multiple flights within a single day, and they had to remain alert for daytime, evening, and nighttime take-offs and landings [41, 42, 45, 48, 62, 67]. Truck drivers had different types of schedules [50, 56, 63]. In the United States, for example, truck drivers were required to take a 34-hr break after accumulating 60–70 hrs of weekly driving. Although this requirement was intended to allow for rest, it led truck drivers’ subsequent assignments to begin and end at different times [63]. For Australian truck drivers who worked in the coal mining industry, however, the work schedules were more predictable because they were assigned to day- or night-shifts (lasting 12-hrs), but their schedules rotated weekly [50].

The Morningness-Eveningness Questionnaire (MEQ; scores range 16–86) was used in four studies, which found that airline pilots and astronauts did not have strong dispositions for being awake or asleep at specific times of the day [44, 45, 58, 66]. For example, the MEQ scores from astronauts [44], short-haul commercial pilots [45], and military pilots [58] indicated that they did not favor early or late sleeping and working times (e.g. MEQ scores of 52.9 ± 12.3 for astronauts, 51.4 ± 7.1 for commercial pilots, and 53.9 ± 8.2 for military pilots). The MEQ was not used in any of the studies about truck drivers [50, 56, 63]. In a stimulated space mission, MEQ scores were used to exclude subjects who reported definite preferences for morningness or eveningness (i.e. study participation required an MEQ score between 42 and 58 to avoid circadian preferences from confounding the study findings) [46].

Circadian biomarkers (e.g. urinary 6-sulfatoxymelatonin levels and 24-hr temperature fluctuations) were analyzed in airline pilots and astronauts [42, 45, 46]. For example, Arsintescu et al. [42, 45] measured airline pilots’ urinary 6-sulfatoxymelatonin rhythms to understand how flight schedules affected their circadian rhythms. They estimated a baseline acrophase (indicating the circadian nadir in each pilot’s 6-sulfatoxymelatonin rhythm) as they flew for 5 consecutive days with mid-morning take-offs. Wide inter-individual circadian phase differences were found among the pilots during this baseline period—the urinary 6-sulfatoxymelatonin acrophases occurred between 0200 and 0630 hrs (*n* = 13). Then, after 3 to 4 days off, the pilots switched to a schedule requiring early-morning take-offs, which was associated with a phase advance in most of the pilots (*n* = 9); however, two pilots had a phase delay—the sample’s acrophases ranged between 0030 and 0450 hrs. When pilots’ schedules rotated (to begin with mid-day take-offs), the sample demonstrated a mean phase advance of 1.3 hrs (*n* = 7 [phase advance]; *n* = 1 [phase delay]). When the pilots’ shifts began in the evening, however, the 6-sulfatoxymelatonin rhythm acrophases occurred between 0200 and 0630 hrs. Although this small sample size limited statistical comparisons, these findings illustrated how pilots were able to acclimate to their schedules [42]. In a study of astronauts, Flynn-Evans et al. [46] tested the hypothesis that space travel would cause a misalignment between the endogenous circadian temperature rhythm and astronauts’ sleep/wake schedules. The Circadian Performance Simulation Software was used to determine how astronauts’ temperatures aligned with their sleep/wake cycles (considering that body temperature rhythms should typically reach a nadir during sleep). A percentage of the astronauts’ temperature rhythms were not aligned with the timing of their sleep/wake periods 11 days before the mission (13%), in space (19%), and on the nights before conducting EVAs (29%). This temperature/sleep timing alignment was an important finding considering that astronauts with a circadian misalignment slept 0.5 fewer hrs and had VAS scores reflecting poorer subjective sleep quality (*p* ≤ .01) compared with astronauts without the misalignment [46]. The temperature/sleep timing and alignment was not significantly associated with astronauts’ subjective reports of fatigue [46].

### Variables affecting performance

To objectively measure performance, subjects completed PVTs lasting 3-, 5-, or 10-min [41, 42, 44–50, 54, 56, 59, 61–63, 66]. Researchers also analyzed performance-related data acquired from the following platforms: NASA Task Load Index (measured mental, physical, and emotional aspects of workload) [41, 53, 62], Reaction Self-Test (a computerized PVT-B that also acquires sleep/wake and workload data) [46], Cognition Test Battery (comprised of 10 neurobehavioral tests designed specifically for astronauts) [59], Pilot Alertness Test [60], and the Robotics On-Board Trainer (ROBoT; NASA’s platform for assessing abilities for docking and grappling maneuvers) [66]. Trucks’ lane deviations and hard-breaking events (signs of inattention and near-collisions) were also analyzed to understand driver performance [56, 63], and a group of ISS astronauts (and healthy control subjects) underwent computerized testing to compare their driving skills (e.g. manual dexterity, dual-tasking, and motion perception) [57].

Signs of degraded performance on the PVT (e.g. slower reaction time [RT], more lapses in attention, and premature responses) were associated with having higher workloads (e.g. higher NASA Task Load Index subscale scores) [41], early-morning start times [45, 53], longer durations of wakefulness [53, 66], shorter sleep before work [45, 53], and elevated fatigue scores (on the KSS, Samn-Perelli scale, or VAS) [41, 42, 45, 48, 49, 53, 60, 62, 64, 65]. Short commercial airline flights (which posed a higher workload for pilots according to the NASA Task Load Index) and early-morning take-offs (that reduced pilots’ sleep duration) were associated poorer performance [41, 45]. For example, comparisons of pilots rotating through schedules with different take-off times revealed how flying earlier in the morning was associated with a slower mean RT and more lapses in attention (257 ± 70 ms [early-morning RT] versus 261 ± 62 ms [mid-day RT], *p* < .01; 4.4 ± 5.4 lapses [number >500 ms; early-morning] versus 4.7 ± 5.1 lapses [mid-day], *p* < .01) [45]. In a flight simulator, pilots also demonstrated significantly more PVT lapses when they had to perform multiple take-offs and landings within a single day (as opposed to a single flight) [53]. The prior night’s sleep duration was associated with the number of PVT lapses and premature responses—each additional hour of sleep reduced the false start rate by 0.4 ± 0.1 (*p* < .001) [53]. In a study of pilots who were flying between the U.S. and Japan, the duration of wakefulness and previous 24-hrs of sleep predicted the slowest 10% RT (at TOD). Every hour of wakefulness increased this PVT metric by 0.05 responses/s, while every additional hour of sleep was associated with an improvement by 0.10 responses/s [47].

The data for truck drivers indicated that off-duty time and rest periods, but not necessarily sleep duration, affected performance. Having >1 night to sleep between jobs was associated with significantly fewer lapses in attention on the PVT-B and fewer lane deviations while driving commercial trucks at night (despite no significant increase in their mean sleep duration during the off-duty period) [63]. A bio-mathematical model determined by Mollicone et al. used truck drivers’ fatigue levels to predict hard-braking events. They determined that each lapse in the PVT increased the drivers’ risk of hard-braking events by 8%, which demonstrates how poorer PVT performance can indicate an elevated risk for accidents [53]. Healthy volunteers also demonstrated performance impairments when they underwent simulated astronaut missions with sleep-restriction and deprivation [44, 57, 59, 66]. When volunteers were sleep-deprived for 28 consecutive hrs before engaging in simulated spacecraft maneuvers, their PVT indicators worsened as they stayed awake (e.g. increase in lapses, slower RT) [66]. In the Human Exploration Research Analog habitat, volunteers’ sleep was restricted to 5 hrs per day (for 5 consecutive nights, followed by 8 hrs of sleep for 2 nights), and their performance was significantly worse during the sleep-restriction period (e.g. slower RT). For astronauts, sleep durations <6 hrs on the ISS were associated with a significantly slower mean RT on the PVT-B [54], and ISS astronauts did not immediately return to their baseline level of performance post-mission. Astronauts required ~4 days post-mission to recover from deficits in motor functioning/perception as a result of space/microgravity (as determined by comparisons against healthy volunteers) [57].

## Discussion

**Figure 1.**
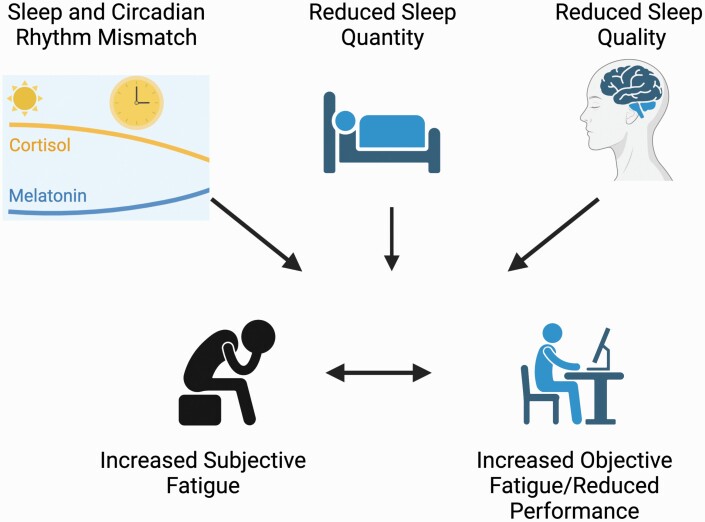
Key mechanisms. Created with BioRender.com

The present review synthesizes findings about sleep, fatigue, and performance in pilots, truck drivers, and astronauts—all three occupations require sustained periods of vigilance to prevent accidents, injuries, and deaths. Our scoping review found that sleep durations <7 hrs (the recommended sleep duration) [1] were common, particularly for truck drivers, astronauts (before and during missions), and pilots with early-morning take-offs. The reviewed studies also demonstrated how longer sleep could significantly improve performance (likely translating into lower risks for errors, collisions, and accidents). Occupational schedules could cause circadian misalignment; therefore, sleep enhancement interventions, fatigue mitigation strategies, and the development of optimal work schedules will be important for pilots, truck drivers, and astronauts (key mechanisms are illustrated in Figure 1).

Upon recognizing how sleep significantly affects job performance, several governing bodies enforced regulations requiring specific break times for sleep in the trucking and aviation industries [22, 39]. Despite the emphasis on breaks, however, the findings of present review illustrate how obtaining ≥7 hrs of sleep remains challenging. It is possible that occupational factors are impairing sleep, such as requirements for working rotating-shifts. Rotating schedules require periods of time when people must sleep during the day, but the schedules’ rotating start and end times make acclimation difficult. Sunlight, an important circadian rhythm regulator, affects neural responses in the anterior hypothalamus, which regulates the pineal gland’s melatonin production [7, 68]. Melatonin levels typically begin rising when lights are dimmed, but shift-work alters the phase of melatonin rhythms. Consequently, shift-workers cannot always adapt their circadian rhythms to their work schedules, especially when schedules are unpredictable, which has been shown in police officers and nurses [17, 69, 70]. These findings emphasize the importance of considering the timing of sleep periods, not only the duration of off-duty time, when designing legislation about workplace health and safety. Complex tasks, learning, memory consolidation, and emotional regulation require adequate sleep. Arsintescu et al. [41] described how pilots’ had higher workload complexities and demands with shorter flights—flight length and sleep duration were both negatively associated with the NASA Task Load Index sub-components, which measured effort, stress, and frustration. These findings illustrate the value of sleep for future endeavors related to workplace satisfaction, training, worker retention, and burnout prevention.

Astronauts’ EVAs illustrate the highest-risk aspects of their work, considering the exposure to dangerous environmental conditions involving extreme cold and hypobaric hypoxia outside of the spacecraft [71, 72]. Despite the dangers, the literature illustrated how astronauts have short sleep (~6 hrs), and they slept even less before EVAs. Considering how space-exploration endeavors are likely to increase in regularity in the future, it will be imperative to develop protocols for ensuring that astronauts can obtain adequate high-quality sleep in space, despite the potential issues caused by weightlessness, changes in atmospheric gases, and the stressors associated with confinement.

Women comprise a minority within the aviation, trucking, and space-exploration workforces, and they were underrepresented in the reviewed studies (Table 1). Only one study specifically addressed the researchers’ attempts to balance the numbers of men and women in their study design [59]. Considering that more women are likely to enter these occupations in the future, it will be important that studies are designed to have adequate statistical power for comparing sleep, fatigue, or performance measures by sex, especially considering previous reports of sex differences in circadian and homeostatic sleep-regulatory factors [73].

In conclusion, future research and policy efforts should focus on developing strategies to increase sleep duration and mitigate fatigue, in addition to advancing knowledge about sleep microstructure and circadian rhythms and their effects on performance abilities. Incorporating the neurobehavioral discoveries from Dinges and colleagues’ [74] research programs into novel prediction models is important for prospectively determining the risks posed by various occupational activities (e.g. EVAs in space, ultra long-haul flights) according to sleep, circadian, genetic, and mission-related predictors to make well-informed decisions about performance and safety.

## Abbreviations

EEG: electroencephalogram
EVA: extravehicular activity
ISS: International Space Station
KSS: Karolinska Sleepiness Scale
MEQ: Morningness-Eveningness Questionnaire
NASA: National Aeronautics and Space Administration
PSQI: Pittsburgh Sleep Quantity Index
PVT: Psychomotor Vigilance Test
PVT-B: Brief Version of the Psychomotor Vigilance Test
ROBoT: robotics on-board trainer
RT: reaction time
TOD: top of descent
VAS: Visual Analog Scale
WASO: wake after sleep onset
